# Single‐Molecule FRET of Membrane Transport Proteins

**DOI:** 10.1002/cbic.202100106

**Published:** 2021-05-21

**Authors:** Kim Bartels, Tanya Lasitza‐Male, Hagen Hofmann, Christian Löw

**Affiliations:** ^1^ Centre for Structural Systems Biology (CSSB) DESY and European Molecular Biology Laboratory Hamburg Notkestrasse 85 22607 Hamburg Germany; ^2^ Department of Structural Biology Weizmann Institute of Science Herzl St. 234 7610001 Rehovot Israel

**Keywords:** FRET, membrane proteins, molecular dynamics, single-molecule studies, transmembrane transporter

## Abstract

Uncovering the structure and function of biomolecules is a fundamental goal in structural biology. Membrane‐embedded transport proteins are ubiquitous in all kingdoms of life. Despite structural flexibility, their mechanisms are typically studied by ensemble biochemical methods or by static high‐resolution structures, which complicate a detailed understanding of their dynamics. Here, we review the recent progress of single molecule Förster Resonance Energy Transfer (smFRET) in determining mechanisms and timescales of substrate transport across membranes. These studies do not only demonstrate the versatility and suitability of state‐of‐the‐art smFRET tools for studying membrane transport proteins but they also highlight the importance of membrane mimicking environments in preserving the function of these proteins. The current achievements advance our understanding of transport mechanisms and have the potential to facilitate future progress in drug design.

## Graphical abstract

**Recent single‐molecule** FRET studies on members of various transporter families reveal the potential of this technique to advance the mechanistic understanding of this protein class.

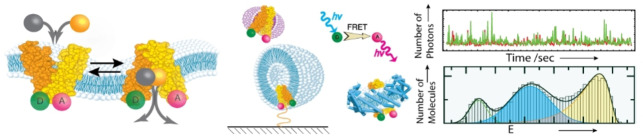

## 1 Introduction

For the last two decades,^[1]^ smFRET techniques have been extensively used to study the properties of molecular machines,^[2]^ intrinsically disordered proteins (IDPs),[^3^, ^4^, ^5^, ^6^, ^7^, ^8^] protein folding processes,[^9^, ^10^, ^11^, ^12^, ^13^, ^14^, ^15^, ^16^] protein‐ligand[^17^, ^18^, ^19^] and protein‐nucleic acid interactions,[^20^, ^21^, ^22^, ^23^] as well as other structure‐function relationships and dynamic processes.[^24^, ^25^, ^26^] SmFRET is a particularly powerful and versatile tool to gain molecular and mechanistic insights because of its high spatial resolution (2–10 nm) combined with a wide range of accessible timescales (ns‐minutes).[^1^, ^10^, ^27^] In fact, smFRET is becoming increasingly popular in the investigation of membrane transporter dynamics (Table 1), thus complementing well‐established methods such as Nuclear Magnetic Resonance (NMR),[^28^, ^29^, ^30^] Small‐Angle X‐ray Scattering (SAXS)[^31^, ^32^, ^33^] and Electron Paramagnetic Resonance (EPR).[^34^, ^35^, ^36^]

**Figure 1:**
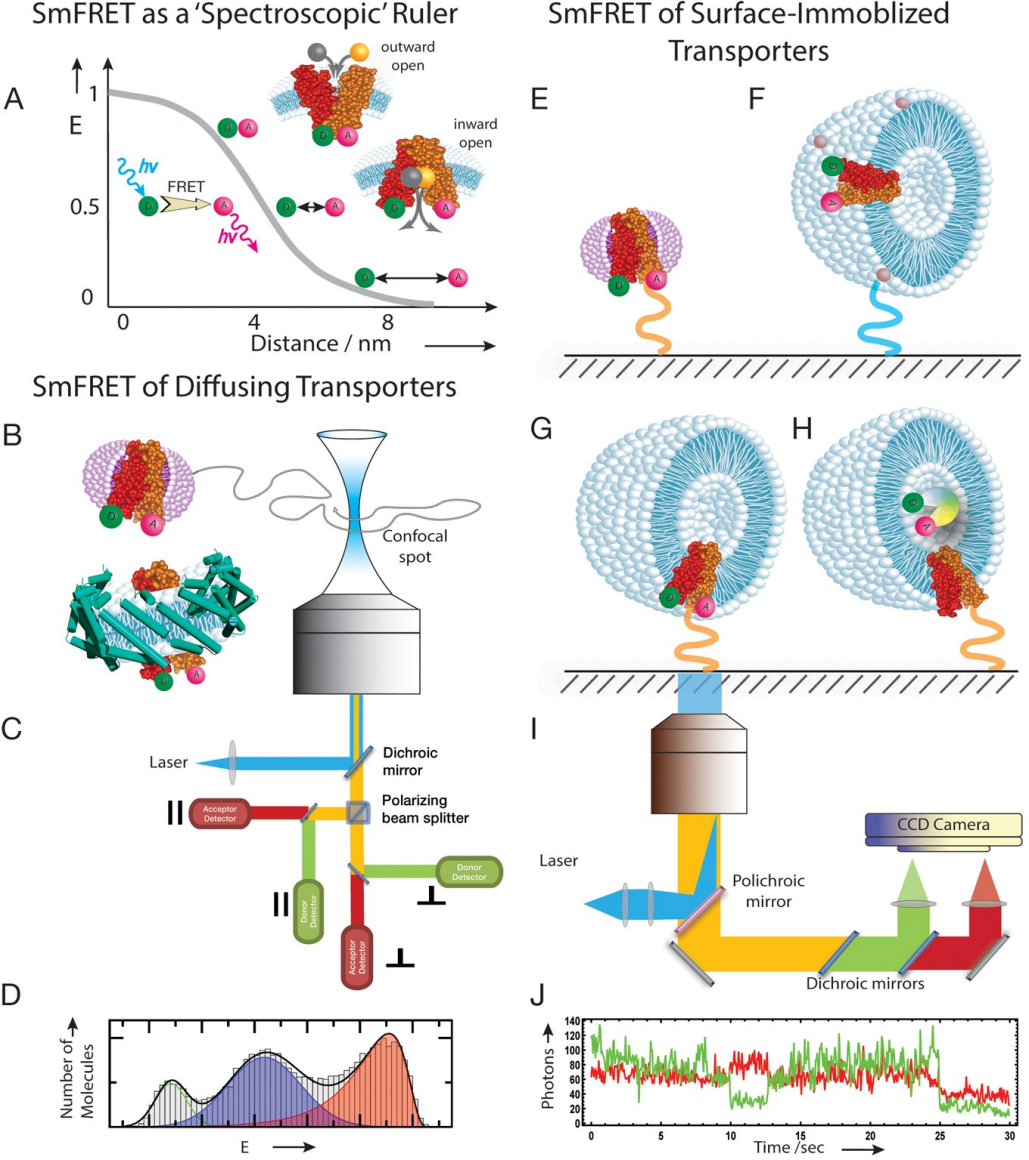
Free diffusing and surface‐immobilized smFRET experiments with membrane transporters. (A) Dependence of FRET efficiency (E) on the distance between donor and acceptor is used as a ‘spectroscopic’ ruler in order to distinguish between outward‐open and inward‐open conformations of the alternating access model. (B) SmFRET of freely diffusing transporters can be performed embedded either in detergent micelles (top) or e. g. in Saposin nanoparticles (bottom). (C) Confocal microscope setup used for smFRET experiments on freely diffusing molecules. (D) Schematic histogram from freely diffusing transporters in detergent micelles. (E) SmFRET of surface‐immobilized transporters in detergent micelles, linked to the surface via the protein. (F) Liposome embedded transporters surface‐immobilized through lipids. (G) Liposome embedded transporters surface‐immobilized through the protein. (H) Liposome embedded wild type transporters, linked to the surface via the protein; the liposomes are supplied with a ligand sensor. The sensor is doubly labeled with a FRET dye pair. (I) TIRF setup used for smFRET experiments with immobilized molecules. (J) Schematic time trace of intramolecular smFRET obtained from a surface‐immobilized single chain doubly labeled transporter in detergent micelles. Green: donor channel, red: acceptor channel. The micelles and liposomes structures were taken from Adobe Stock.

In FRET, the excitation energy of a donor fluorophore (D) is transferred to an acceptor fluorophore (A) in a non‐radiative manner (Figure 1A).^[37]^ The efficiency of the energy transfer (E) depends sensitively on the distance *r* between D and A via $E=R_{0}^{6}/\left(R_{0}^{6}+r^{6}\right)$ where $R_{0}\;$ is the Förster distance, which is characteristic for each D−A pair and defines the range of distances that can be probed.^[38]^ For common dyes such as cyanine or rhodamine derivatives, Förster distances are in the order of a few nanometers, which makes the method ideal for studying conformational changes in proteins. FRET as a tool to study protein conformations and dynamics became particularly powerful with the advent of single‐molecule detection.[^39^, ^40^] Overcoming ensemble averaging by determining FRET‐efficiencies in individual molecules^[41]^ opened the door to distinguish alternative conformations and their interconversion kinetics within the same protein.[^42^, ^43^, ^44^] Currently, two main approaches are typically used in smFRET data acquisition:^[2]^ (i) observation of freely diffusing molecules in solution,[^18^, ^45^] and (ii) monitoring of molecules that are immobilized on a surface[^46^, ^47^] (Figure 1B–J).

The observation of freely diffusing molecules provides information about conformational dynamics at timescales from nanoseconds to milliseconds but it is limited by the diffusion time of the molecule through the confocal spot (∼1 ms).^[10]^ Yet, this limitation has recently been extended to timescales up to 20 ms using Recurrence Analysis of Single Particles (RASP).[^48^, ^49^] RASP exploits the fact that at low picomolar concentrations, a freely diffusing molecule has a higher probability to return to the confocal volume than a new molecule to enter the volume. If the timescale of dynamics is within the recurrence time of a molecule, snapshots of different conformations of the molecule can be observed.^[48]^ Faster dynamics (<1 ms) are typically obtained from fluorescence correlation spectroscopy (FCS) in combination with smFRET,[^50^, ^51^] Probability Distribution Analysis (PDA),[^52^, ^53^] adapted Hidden‐Markov‐Models (H2MM),^[54]^ or multi‐parameter photon‐by‐photon analysis[^52^, ^55^] of freely diffusing molecules.

An elegant approach to extend the timescales of smFRET experiments to seconds and even minutes, is the surface immobilization of molecules or their encapsulation in immobilized vesicles.[^12^, ^56^, ^57^, ^58^] Usually, trajectories are collected with a camera in a total internal reflection (TIRF) microscope (Figure 1I), which provides single‐molecule trajectories of donor and acceptor intensities for many molecules in one experiment. As a downside, time resolution is limited by the frame rate of the camera, which is typically>10 ms such that faster dynamics often remain inaccessible.^[27]^ Alternatively, confocal microscopes with substantially higher time resolution can be used, albeit with the trade‐off that individual molecules have to be monitored one at a time.^[1]^

Although membrane proteins play a key role in cell metabolism: they are encoded by roughly 30 % of the human genome[^59^, ^60^] and account for an estimated 60 % of drug targets,^[61]^ a full understanding of the interplay between conformational dynamics and their function is largely elusive. Among these proteins, transporters constitute a large class of integral membrane proteins.

Transporters are typically classified based on the direction and type of transport.^[62]^ Passive transporters move substrates along a concentration gradient (facilitative diffusion) whereas substrates are moved against their concentration gradient by active transporters.^[63]^ Active transport requires energy to transport substrates against their chemical potential gradients. They are therefore subdivided based on the energy source used for this “uphill” transport. Primary active transporters use the hydrolysis of ATP whereas secondary active transporters couple substrate transport to pre‐established electrochemical gradients of ions across the cell membrane such as protons, sodium, potassium, or chlorine.[^63^, ^64^, ^65^]

**Figure 2:**
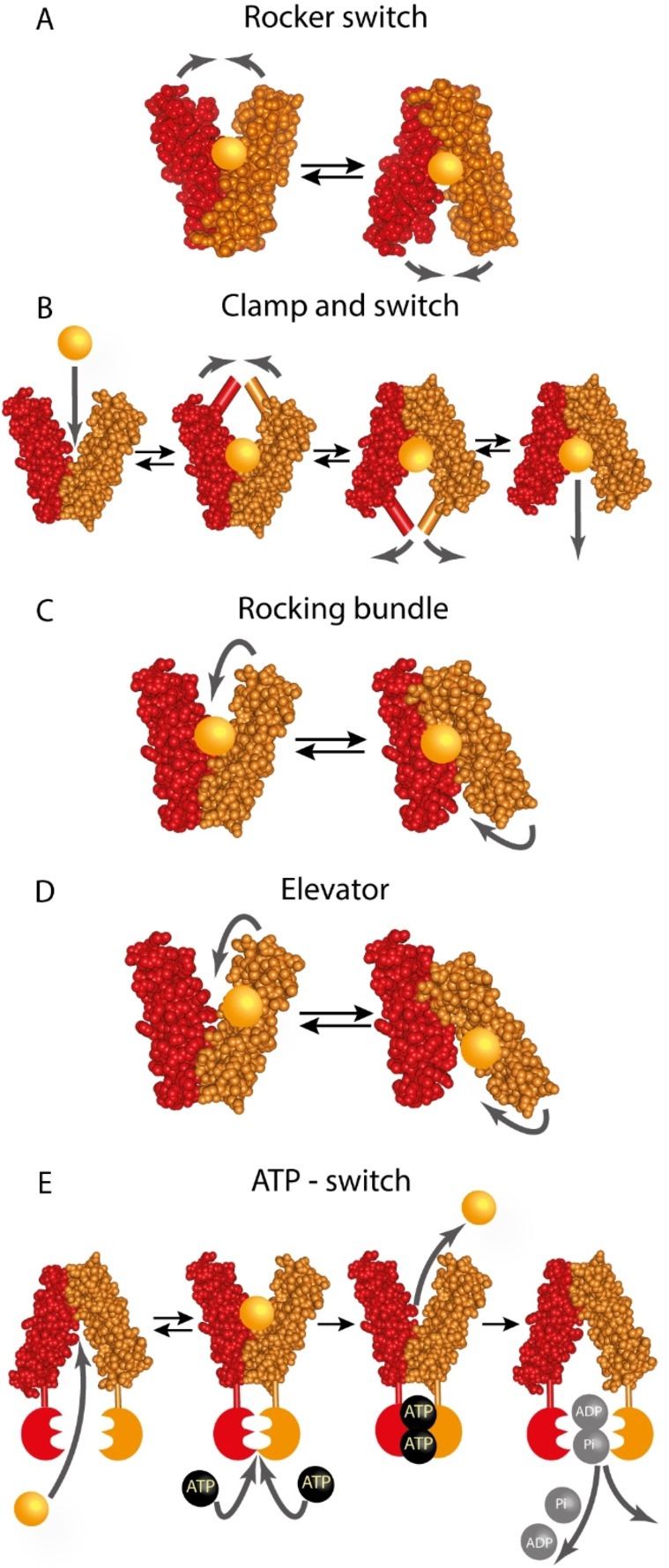
Alternating access mechanisms schematic representation. (A) Major conformations of the rocker switch mechanism. The substrate binds to the binding site in the outward open state, resulting in a rearrangement of the domains to form the inward open state, where the substrate is released into the cell. (B) Schematic representation of the clamp and switch model, which is a modified version of the rocker switch and rocking bundle mechanism. The model includes the bending of particular transmembrane helices throughout the transport cycle and covers outward‐facing occluded and inward‐facing occluded states. (C) Rocking bundle model (“gated‐pore model”). The binding site is located between both domains as described for the rocker switch model, but the rearrangements of the domains around the binding site are not symmetrical. (D) The elevator model. In contrast to the (A)‐(C) models, the substrate binding site is located only on one of the transporter's domains. The domain that binds the substrate is moving against an immobile structurally different domain and physically translocates the substrate into the cell. (E) Schematic representation of the ATP‐switch model for primary active exporters. The transported substrate first binds to the binding site in the transmembrane domains (TMDs), which results in a conformational change of the nucleotide binding domains (NBDs) that now have an increased affinity for ATP. The closed NBD dimer induces a conformational change in the TMDs such that the substrate binding site is exposed extracellularly and its affinity is reduced, leading to substrate release. Afterwards, ATP is hydrolyzed, ADP and Pi release restores the transporter to its basal conformation. The figure is adopted from references [66–68].

In general, transporters are predicted to work by an alternating‐access model^[69]^ in which the binding site is alternatingly exposed to either side of the membrane. Different transporter families follow different transport mechanisms to achieve alternating access (Figure 2). Currently proposed transport models are “rocker switch” for Sugar‐Will‐Eventually‐be‐Exported‐Transporters (SWEETs) or “clamp and switch” for Major‐Facilitator‐Superfamily (MFS) transporters, “rocking bundle” for the Neurotransmitter‐Sodium‐Symporters (NSS) family and the “elevator” mechanism for members of the Excitatory‐Amino‐Acid‐Transporters (EAAT) family.[^67^, ^70^, ^71^, ^72^, ^73^, ^74^] For primary active ABC transporters, the “ATP‐switch model” has been described.^[75]^

## 2 Challenges of Studying Transporters with smFRET and How to Overcome Them

### 2.1 Specific labeling and functionality

An efficient and widely used way to selectively label proteins uses maleimide‐thiol chemistry that specifically targets cysteine residues.[^44^, ^76^, ^77^] This often requires the exchange of native cysteine residues to serine, alanine, or to other amino acids found in homologous transporters.^[78]^ Importantly, not all intrinsic cysteine residues necessarily need to be replaced.^[79]^ An accessibility test with PEG‐maleimide for instance, can outline non‐accessible cysteines that would not interfere with the site‐specific labeling of cysteines of interest.^[80]^ An alternative labeling strategy uses noncanonical amino acids,[^81^, ^82^] thus providing an orthogonal labeling chemistry^[83]^ that is particularly advantageous in combination with cysteine‐maleimide labeling. Alternatives are tag‐based labeling approaches such as used for the dimeric G‐protein‐coupled‐receptor (GPCR) mGluR1. Here one monomer was fused to a CLIP‐tag and the other to a SNAP‐tag.^[84]^ SNAP‐ and CLIP‐tags are protein‐labeling tools that allow a site‐specific and covalent attachment of fluorescent dyes. The tags are based on the small DNA repair protein O^6^‐alkylguanine‐DNA‐alkyltransferase. The significant size of these tags (182 aa) is often compensated by a high labeling selectivity.^[85]^ However, care has to be taken when chemically modifying the transporter under investigation. Even if large labeling tags are avoided, the fluorescent dyes used in smFRET experiments need to obey high quantum yields, good photo‐stability, with excitation and emission wavelengths in the visible range of the spectra, thus narrowing choices to rhodamine and cyanine derivatives.[^86^, ^87^] These dye families are often bulky and hydrophobic and therefore risk impaired functions via unspecific interactions with the protein. For example, although the dye Atto647N is a bright and photo‐physically robust dye, it has been shown to interact with hydrophobic protein patches,^[88]^ which renders it less suitable, particularly for membrane proteins. In general, dyes and labeling positions need to be carefully chosen. As a general rule, strongly hydrophobic dyes and solvent‐inaccessible regions of proteins should be avoided to minimize dye‐protein interactions. Dyes used successfully in smFRET‐studies of membrane transporter proteins are listed in Table 1. Yet, even if these requirements are met, activity assays should be used to confirm the functional integrity of the transporter after mutation and labeling. These assays include antibodies that recognize structural epitopes,^[89]^ thermal unfolding assays of the protein in the presence and absence of ligands,^[89]^ binding assays *via* bilayer interferometry^[89]^ or Surface Plasmon Resonance^[90]^ or other biophysical and biochemical techniques.[^90^, ^91^, ^92^, ^93^, ^94^, ^95^, ^96^] The impact of mutations on function can be monitored by ATPase assays[^79^, ^90^, ^97^, ^98^, ^99^, ^100^, ^101^] or transport assays.[^78^, ^90^, ^93^, ^94^, ^96^, ^97^, ^100^, ^102^, ^103^, ^104^, ^105^, ^106^, ^107^, ^108^] In transport assays, the protein is either overexpressed in a cell or incorporated into liposomes and the accumulation of fluorescent[^78^, ^97^, ^105^] or radiolabeled substrate[^90^, ^93^, ^94^, ^96^, ^100^, ^102^, ^103^, ^106^, ^107^, ^108^] in the compartment (cell or vesicle) is measured. Good examples for such careful controls are LmrP transporter variants whose activities were checked with the fluorescent ligand Hoechst,^[105]^ or variants of the Glt_Ph_ transporter in liposomes where the uptake of the radioactive substrate [^3^H]‐Asp was used to confirm activity.^[108]^

**Table 1:** Summary of smFRET studies conducted on membrane transporters.^[a]^

Protein name	Source organism	Protein family	SmFRET regime	Labeling strategy	Protein environment	FRET‐dye pair	Optical tools and methods	Ref.
AtSWEET	*Arabidopsis thaliana*	SWEET	Diffusion in micelles	Cysteine maleimide	Detergent (DM)	Cy3/Cy5	TIRF	[78]
bcMAlT	*Bacillus cereus*	EIIC	Diffusion in micelles	Cysteine maleimide	Detergent (DDM)	Alexa488/Alexa594	Confocal	[102]
BetP	*Corynebacterium glutamicum*	BCCT	Diffusion in micelles	Cysteine maleimide	Detergent (DDM)	Alexa555/Alexa647 vs Cage552 and Cy5 with reducing agents	Confocal	[103]
BtuCD	*E. coli*	ABC	Immobilized in micelles and liposomes *via* a tag on the transporter	Cysteine maleimide	Detergent (LDAO), liposomes (*E. coli* lipids/PC 3 : 1 w/w)	Cy3/Cy5	TIRF	[90]
CLC‐ec1	*E. coli*	CLC	Immobilized in micelles *via* a tag on the transporter	Cysteine maleimide	Detergent (DM)	Alexa555/Alexa647	Confocal	[104]
DtpA	*E. coli*	MFS	Diffusion in micelles and SapNPs	Cysteine maleimide	Detergent (LMNG, DDM), SapNPs (POPA, POPE, POPS, Brain lipid total extract)	Alexa488/Alexa594	Confocal	[89]
EmrE	*E. coli*	SMR	Immobilized *via* a tag on the bicelle lipid	Cysteine maleimide	Bicelles (DLPC/DHPC, DMPC/DHPC)	Cy3/Cy5	TIRF	[181]
Glt_Ph_	*Pyrococcus horikoshii*	EAAT	Immobilized in micelles *via* a tag on the transporter	Cysteine maleimide	Detergent (DDM)	Cy3/Cy5	TIRF	[108]
			Immobilized in liposomes *via* a tag on the transporter		Liposomes (*E. coli* lipids/PC 3 : 1 w/w)			[107]
			Immobilized *via* a tag on the liposome lipid		Liposomes (w/w 40 % DOPC, 29 % DOPE, 30 % DOPG, 1 % biotin‐DOPE)	Alexa555/Alexa647	TIRF	[114]
			Immobilized in liposomes *via* a tag on the unlabeled transporter	Cysteine maleimide labeling of the sensor protein	Liposomes (*E. coli* lipids/PC 3 : 1 w/w)	LD555p/LD655	TIRF	[106]
LacY	*E. coli*	MFS	Diffusion in micelles	Cysteine maleimide	Detergent (DDM)	Alexa488/Alexa647	Confocal	[91]
LCMA1	*Listeria monocytogenes*	P‐type ATPase	Immobilized in micelles *via* a tag on the transporter	Cysteine maleimide	Detergent (C_12_E_8_)	LD550/LD650	TIRF	[79]
LeuT	*Aquifex aeolicus*	NSS	Immobilized in micelles *via* a tag on the transporter	Cysteine maleimide	Detergent (DDM)	Cy3/Cy5	TIRF	[93,94]
						Cy3/Cy5 and LD550/LD650		[95]
						LD550/LD650		[96]
LmrP	*Lactococcus lactis*	MFS	Diffusion in micelles	Cysteine maleimide	Detergent (DDM)	ATTO488/Alexa647	Confocal	[105]
McjD	*E. coli*	ABC	Diffusion in micelles, and immobilized *via* a tag on the liposome lipid	Cysteine‐maleimide	Detergent (DDM), liposomes (*E. coli* polar lipids, DOPE/DOPG/biotin‐DOPE)	Alexa555/Alexa647	Confocal	[97]
MdfA	*E. coli*	MFS	Immobilized in micelles *via* a tag on the transporter	Cysteine maleimide	Detergent (DDM)	Cy3/Cy5	TIRF	[92]
MhsT	*Bacillus halodurans*	NSS	Immobilized *via* a tag on the liposome lipid	Cysteine maleimide labeling of the sensor protein	Liposomes (*E. coli* lipids/PC/DSPE‐PEG‐maleimide lipids 3 : 1:0.01 w/w)	LD550/LD650	TIRF	[110]
MRP1	*Bos taurus*	ABC	Immobilized in micelles *via* a tag on the transporter	CoA conjugates	Detergent (digitonin)	Cy3/LD655	TIRF	[99]
MsbA	*E. coli*	ABC	Immobilized in micelles *via* a tag on the transporter, and immobilized in nanodiscs *via a* nanodisc lipid	Cysteine maleimide	Detergent (DDM), liposomes (*E. coli* total lipid extract), nanodiscs (*E. coli* total lipid extract/biotin‐PE)	Cy3/Cy5	TIRF	[98]
OpuA	*Lactococcus lactis*	ABC	Diffusion in micelles, and immobilized in micelles and nanodiscs *via* a tag on the transporter	Cysteine maleimide	Detergent (DDM), nanodiscs (DOPC/DOPE)	TMR/Cy5, Alexa555/Alexa647	Confocal/TIRF	[100]
OxlT	*E. coli*	MFS	Immobilized in liposomes *via* a tag on the transporter	Cysteine maleimide	Liposomes (DHPC/ *E. coli* polar lipid extract)	Cy3/Cy5	Confocal	[162]
PgP	*Mus musculus*	ABC	Diffusion in liposomes	Cysteine maleimide	Liposomes (PC/PA)	Alexa488/ATTO610	Confocal	[101]

[a] *N*,*N*‐Dimethyldodecylamine N‐oxide (LDAO), *n*‐dodecyl‐β‐D‐maltoside (DDM), ), *n*‐decyl‐β‐D‐maltoside (DM), lauryl maltose neopentyl glycol (LMNG), 1,2‐dioleoyl‐sn‐glycero‐3‐phosphate (PA), phosphatidylcholine (PC), phosphatidylethanolamine (PE), 1‐palmitoyl‐2‐oleoyl‐sn‐glycero‐3‐phosphate (POPA), 1‐palmitoyl‐2‐oleoyl‐sn‐glycero‐3‐phosphoethanolamine (POPE), 1‐palmitoyl‐2‐oleoyl‐sn‐glycero‐3‐phospho‐L‐serine (POPS), 1,2‐diheptanoyl‐sn‐glycero‐3‐phosphocholine (DHPC), 1,2‐dilauroyl‐sn‐glycero‐3‐phosphocholine (DLPC), 1,2‐dimyristoyl‐sn‐glycero‐3‐phosphocholine (DMPC), 1,2‐dioleoyl‐sn‐glycero‐3‐phosphoethanolamine (DOPE), 1,2‐dioleoyl‐sn‐glycero‐3‐phosphocholine (DOPC), 1,2‐dioleoyl‐sn‐glycero‐3‐phospho‐(1′‐rac‐glycerol) (DOPG), octaethylene glycol monododecyl ether (C12E8), 1,2‐distearoyl‐sn‐glycero‐3‐phosphoethanolamine (DSPE).

**Figure 3:**
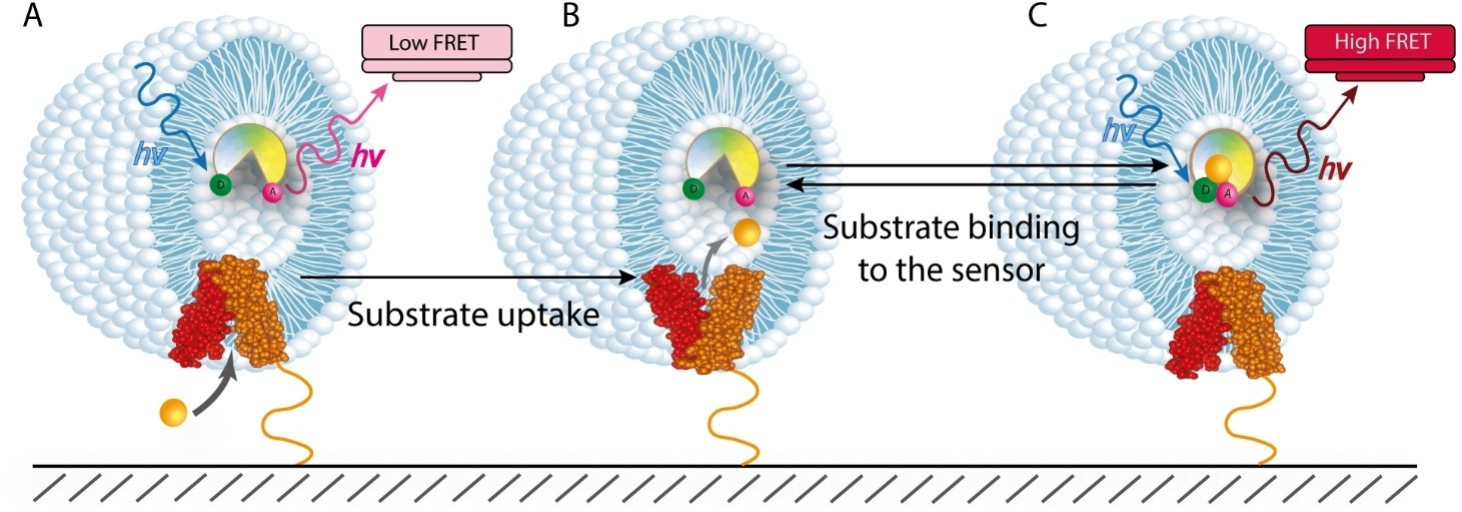
FRET labelled substrate sensor protein is used to measure wild type transporter activity. (A) The transporter (red and orange) is reconstituted into the liposome membrane, while the FRET labelled sensor is trapped inside the liposome and produces low FRET values in the absence of a substrate. (B) A substrate (yellow ball) is taken up by the transporter and released to the lumen of the liposome. (C) The transported substrate binds to the sensor protein and induces a conformational change leading to a high‐FRET state, compared to the apo state of the sensor.

An elegant tool to fully avoid direct protein modifications are smFRET sensors that selectively probe the presence of substrates in vesicles (Figure 3).^[109]^ Here, a single unlabeled wild type transporter molecule is reconstituted into a liposome membrane. To probe transport, FRET‐labeled sensor proteins are simultaneously trapped inside the liposome and alter their conformation upon substrate binding, thus causing a change in FRET efficiency. Whereas this method of monitoring transport is certainly minimally invasive, it also requires calibrations. Parameters such as the affinity of the sensor for its ligand, the sensor concentration, ligand‐sensor association rates to determine the response time of the sensor, and liposome sizes are required to precisely measure the transport rate of single transporters. A challenge of this method is certainly the identification and availability of ligand sensor proteins, but various examples have been described.[^106^, ^110^] Periplasmic binding proteins (PBPs) labeled with a donor‐acceptor pair are frequently used as smFRET sensors. These proteins are known to work *via* a “Venus‐flytrap” mechanism,[^111^, ^112^, ^113^] i. e., substrate binding induces a large conformational change in the sensor that is monitored with smFRET. Labelled PBPs were encapsulated inside liposomes with membrane‐reconstituted wild type transporters and substrate‐uptake by the unlabeled transporter could be followed *via* changes in the FRET efficiency of the PBP. However, engineering a sensor for a given substrate is rather difficult and given the broad variety of ligands that are transported by different transporter families, smFRET‐sensors are not yet broadly applicable.

A particular challenge has always been the investigation of large proteins and oligomeric transporters. However, recent advances show that the strategic labeling of oligomeric transporters can even be an advantage.[^107^, ^108^, ^114^, ^115^]

A particularly successful example is the homo‐trimeric Glt_Ph_ transporter. Here, each subunit was labeled with either a donor, an acceptor, or a surface‐binding tag.^[108]^ After assembly of the trimer, only complexes in which at least one subunit contained a binding tag could be immobilized. Molecules with more than two FRET labels were washed off such that only doubly labeled trimers remained bound to the surface. This strategy also proved to be useful for the reconstitution of Glt_Ph_ transporters in liposomes. Only molecules of the desired orientation, i. e., with the surface‐binding tag on the outside of the liposomes were immobilized whereas molecules that were inserted in the opposite direction were washed off the surface.^[107]^

Another example of smFRET experiments with oligomeric transporters is described in the recent study of the dimeric Cl^−^/proton exchanger CLC‐ec1 from *E. coli*, where the dynamics of the individual subunits was explored.^[115]^ In this dimer, one of the subunits was labeled with a fluorescent dye pair whereas the other subunit was modified with a surface‐binding tag. The transporter was trapped in an inactive conformation *via* point mutations in the gating motifs. After the self‐assembly of the monomers into dimers, only CLC‐ec1 molecules with at least one inactive and surface‐binding monomer were immobilized and only those dimers with a labeled second monomer were detectable in the smFRET experiments. Thus, all dimers contained one active and one inactive monomer, which allowed the authors to selectively monitor the dynamics of only one monomer in the assembly.

A distinct approach for smFRET experiments with an oligomeric protein was used in another study on Glt_Ph_.^[114]^ Here, liposomes were immobilized *via* biotinylated lipids and the subunits of the transporter were labeled with donor and acceptor dyes in a 1 : 2 or 2 : 1 ratio. Afterwards, the different species were separated on the basis of the expected dye‐stoichiometries, using the Alternating‐Laser‐Excitation (ALEX) method.^[116]^

A proof‐of‐concept study on the novel concept of “caging chromophores”^[117]^ by reductive agents or activation upon UV‐light application was recently demonstrated for the trimeric BetP^[103]^ transporter by Jazi *et al*. The combination with ALEX^[118]^ was then used to resolve interactions of multi‐subunit proteins labeled with more than two dyes, thus providing additional possibilities of studying oligomeric proteins by smFRET tools.

### 2.2 Membrane mimicking environment

An important factor in the study of transporters is the membrane environment, which is often difficult to realize experimentally.[^119^, ^120^] Detergent micelles are commonly used for solubilization and stabilization of membrane proteins in structural biology.^[121]^ Thus numerous smFRET studies have been performed using detergents[^121^, ^122^, ^123^] and provided important insights.[^78^, ^79^, ^91^, ^92^, ^93^, ^94^, ^99^, ^102^, ^104^, ^105^, ^108^] Yet, studies of transporters in membrane environments, which better mimic native conditions, showed that lipids play important roles in maintaining their structural and functional integrity.[^120^, ^124^, ^125^, ^126^, ^127^, ^128^]

For certain transporters such as Glt_Ph_, dynamics in micelles do not differ significantly from those found in a lipid bilayer.[^107^, ^108^] For other transporters such as DtpA, the lipid composition has been found to tune the abundance of different conformational states.^[89]^ This study showed that it is not only important to mimic the presence of a membrane, but ideally also its composition. Lipid membrane imitation is usually achieved *via* liposomes or nanodiscs/nanoparticles.^[129]^ Clearly, liposomes are favored since they permit substrate and/or pH gradients that can be used to monitor the incorporated transporter while it performs the designated function.[^93^, ^94^]

However, creating liposomes of complex lipid composition^[130]^ and efficiently incorporating transporters^[131]^ is challenging. In such cases, nanodiscs and nanoparticles are valuable alternatives.[^132^, ^133^, ^134^] Although these assemblies do not permit chemical gradients, they can be used to study the potential influence of the lipid composition on the transport cycle.[^135^, ^136^]

## 3 Recent smFRET Studies on Transporters

**Figure 4:**
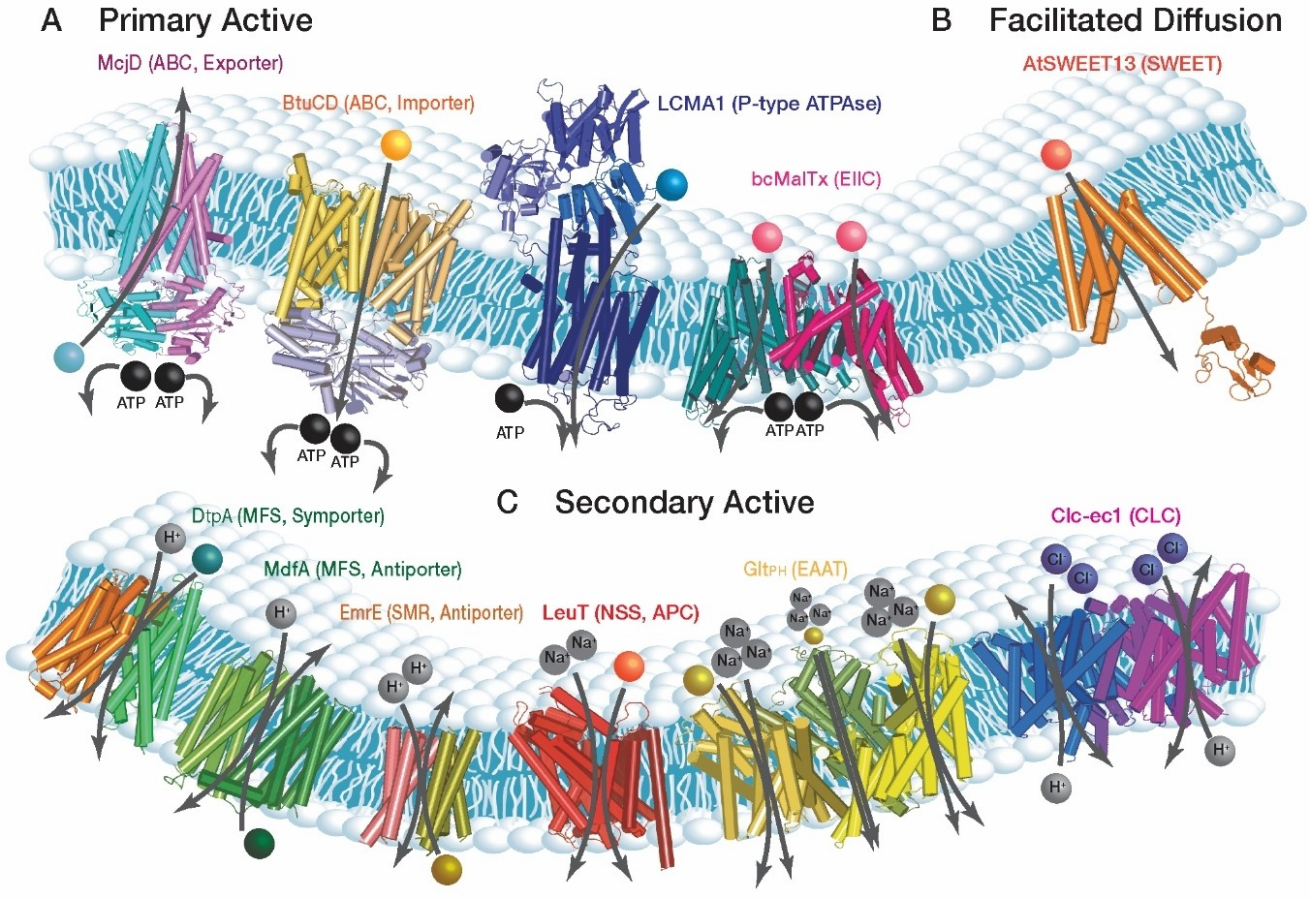
Transporter families explored by smFRET. (A) Primary active transporters (left‐right): McjD (*E. coli*) antimicrobial peptide exporter, ABC family (PDB entry 4PL0), BtuCD (*E. coli*) vitamin B12 importer, ABC family (PDB entry 1L7V), LCMA1 (*Listeria monocytogenes*) Ca^2+^ transporter, P‐type ATPases family (PDB entry 1T5T), bcMalTx (*Bacillus cereus*) sugar uptake, EIIC family (PDB entry 6BVG). (B) Facilitative diffusion transporter: AtSWEET13 (*Arabidopsis thaliana*) mono‐ and disaccharides bi‐directional transporter, SWEETs family (PDB entry 5XPD). (C) Secondary active transporters (left‐right): DtpA (*E. coli*) proton‐dependent oligopeptide transporter, MFS family (PDB entry 6GS4), MdfA (*E. coli*) multi‐drug proton antiporter, MFS family (PDB entry 4ZOW), EmrE (*E. coli*) poly‐aromatic cation substrate coupled to proton antiporter, SMR family (PDB entry 2I68), LeuT (*Aquifex aeolicus*) sodium dependent leucine symporter, APC superfamily (PDB entry 2 A65), Glt_Ph_ (*Pyrococcus horikoshi*) sodium coupled aspartate transporter, EAAT family (PDB entry 2NWX), CLC‐ec1 (*E. coli*) proton‐coupled chlorine antiporter, CLC family (PDB entry 4KK6).

SmFRET has already been used to study transporters from various families. Representatives for each family and their respective substrates are illustrated in Figure 4. Some transporters such as LeuT[^93^, ^94^, ^95^, ^96^] and Glt_Ph_[^106^, ^107^, ^108^, ^114^] were subject of several studies while others such as AtSWEET13,^[78]^ bcMAlT^[102]^ and ClC‐ec1^[104]^ were studied less frequently. In the following, we summarize the results obtained for the different transporter families and highlight individual examples.

### 3.1 ABC transporter family

ATP binding cassette (ABC) transporters are primary active transporters.^[137]^ The prokaryotic members of this family can be importers or exporters while only exporters are found in eukaryotes.^[138]^ These transporters use ATP hydrolysis to power the transport of a vast variety of substrates across the membrane.^[139]^ ABC transporter consist of two transmembrane domains (TMDs) each being linked to a nucleotide binding domain (NBD). They can be encoded by a single polypeptide chain or assemble into homodimers.^[138]^ Furthermore, heterodimeric ABC transporters are also commonly found.^[140]^

In addition, importers possess PBPs that bind the substrate and deliver it to the transmembrane transporter.[^138^, ^141^] Numerous FRET studies investigated isolated PBPs and their interaction with the TMDs, which were reviewed elsewhere.^[142]^

SmFRET studies were carried out on prokaryotic (MsbA, McjD, BtuCD from *E. coli* and OpuA from *Lactococcus lactis*) and eukaryotic (PgP from *Mus musculus* and MRP1 from *Bos Taurus*) ABC transporters.[^90^, ^97^, ^98^, ^99^, ^100^, ^101^] Out of these, OpuA and BtuCD are characterized as importers whereas all others are exporters.

These studies revealed different conformations of the NBDs, at least three in the case of the multidrug‐efflux protein PgP and five for MRP1, the latter being a transporter that expels toxic compounds from the cell and confers resistance to anticancer drugs.[^99^, ^101^] In MRP1, four of the five conformations were assigned to inward‐open states of the transporter and one to the outward‐open state.^[99]^ For PgP, residence times in its different conformational states were in the order of tens of milliseconds in the absence of substrate, which is faster than those found for McjD (>100 ms).[^97^, ^101^] Hence, although these transporters belong to the same family, their dynamics cover different timescales. Under transport conditions, i. e., in the presence of substrates such as cyclosporin and verapamil, the dynamics generally accelerated albeit not for all kinetic steps.^[101]^ For instance, substrate‐binding accelerated the transition from inward‐open to the outward‐open state for MRP1 but it had no effect on the reverse reaction.^[99]^ Interestingly, the oligomerization state and the conformation of MRP1 are also sensitive to the presence of a substrate. For instance, ATP‐binding induces dimerization of the NBDs and shifts the transporter to the outward‐open conformation, a state that was stable for more than 20 s under saturating ATP conditions.^[99]^

Of all investigated ABC exporters, only McjD and MsbA were studied both in detergent and in membrane environments (liposomes and/or nanodiscs).[^97^, ^98^] These studies revealed that both transporters are sensitive to their environment although they do not necessarily exhibit the same conformational changes under comparable conditions. For example, ATP binding and substrate transport are decoupled for MsbA in detergent,^[98]^ which had not been observed for other ABC transporters. In addition, MsbA can switch from a closed to an open state in the presence of ATP,^[98]^ whereas this switch requires both ATP and substrate binding for McjD.^[97]^

A careful comparison showed that the data for McjD are overall consistent between detergent and a lipid environment with only minor differences. The opening of the TMDs in the presence of substrate is slightly wider and the closure of the NBDs with ATP is less tight in liposomes compared to detergent.^[97]^ A stronger impact of the environment was observed for MsbA.^[98]^ Here, even differences between liposomes and nanodiscs were found. Whereas the transport cycle lasted 0.1 s^−1^ or longer in nanodiscs, the protein was more dynamic in liposomes.^[98]^

In contrast to exporters, no spontaneous fluctuations of the NBDs and TMDs were observed for the importer BtuCD. Yet, the time‐resolution in these experiments was 50 ms and faster dynamics may have been missed.^[90]^ Similar to the exporters, the oligomerization state and the conformation of BtuCD was sensitive to ATP. However, another substrate, vitamin B12, did not influence the conformational state of the transporter.^[90]^ Analogously to McjD, BtuCE behaved similar in detergent and nanodiscs with the exception that the FRET changes were larger in nanodiscs compared to detergent, suggesting a higher flexibility of the transporter and again highlighting the importance of a lipid environment.^[90]^

Finally, we would like to note that homology modeling had been successfully used to design labeling positions as shown for OpuA.^[100]^ In general, the strength of smFRET in elucidating dynamic processes benefits strongly from structure‐determination methods such as x‐ray crystallography or cryo‐electron microscopy.

### 3.2 P‐type ATPase family

Another well‐studied family of primary active transporters are Phosphorylation‐type (P‐type) ATPase transporters. These membrane proteins are found in all kingdoms of life where they pump cations against their concentration gradients utilizing ATP hydrolysis, which preserves the electrochemical potential across cell membranes.^[143]^ Although structures of several conformational states along the transport cycle have been reported,[^144^, ^145^, ^146^, ^147^, ^148^, ^149^, ^150^, ^151^, ^152^] it could not be excluded that additional intermediate states exist. Traditionally, the catalytic pathways and the kinetic rates that lead to the formation of intermediate conformations were studied with biochemical ensemble methods that focused on isolated partial reactions of the transport cycle.[^153^, ^154^, ^155^, ^156^] These results suggested a common mechanism for P‐type transporters^[152]^ but they also spotted ambiguities in the rate limiting steps. A recent smFRET study aimed at resolving these ambiguities. The experiments monitored the entire transport cycle of the prokaryotic Ca^2+^‐ATPase ortholog LCMA1 from *Listeria monocytogenes* ^[79]^ and indicate that the steps preceding the formation of the so‐called E1P‐state, in which the transporter is bound to ATP and Ca^2+^, are rate limiting. In addition, smFRET allowed to monitor so‐called de‐occluded conformations that couple ion transport and ATP hydrolysis. Unfortunately, these states have not yet been described by x‐ray crystallography or other imaging techniques to date.

### 3.3 EIIC family

Membrane embedded Enzymes IIC (EIIC) are essential components of the phosphoenolpyruvate‐dependent phosphotransferase system that are responsible for sugar uptake. In complex with EIIC‐transporters, sugar is phosphorylated and dissociates.^[157]^ For EIIC enzymes, the switch between the outward‐open and inward‐open conformations was suggested to occur through elevator‐like movements of the subunits.^[157]^ Yet, only the outward‐open conformation had been crystallized before for the bcMAlT‐transporter from *Bacillus cereuss*.^[157]^ Ren *et al*. crystallized the inward‐open conformer in a cross‐linked form and utilized smFRET to verify that this form is indeed similar to the native conformation.^[102]^

Further smFRET experiments on the native transporter without the crosslinker, identified two conformations in detergent solution out of which the outward‐open conformation was thermodynamically favored, which explains the crystallization bias towards this conformer.^[102]^

### 3.4 MFS

The Major Facilitator Superfamily (MFS) is a superfamily of secondary active transporters that is ubiquitous in all kingdoms of life.^[158]^ It includes uniporters, symporters, and antiporters.^[159]^ Initially described as sugar transporters, they were recognized to transport a diverse range of substrates.^[160]^

All MFS transporters share a common fold. It is characterized by 12 transmembrane helices (TMs) that are grouped into two bundles of six consecutive helices each. The bundles are referred to as the N‐terminal and C‐terminal bundle and enclose the substrate binding site.^[160]^ The current transport model proposes that MFS transporters work *via* an alternating access mechanism.[^69^, ^161^]

The first smFRET experiment on an MFS transporter was carried out on OxlT, an Oxalate:formate antiporter from *E. coli* ^[162]^ that was reconstituted into surface‐immobilized liposomes. The observed FRET efficiency agreed well with the crystal structure. A broadening of the histogram was observed but due to high background noise, it was not possible to conclude that this resulted from the presence of several conformational states in the sample.^[162]^ However, another smFRET study has linked such peak broadening to the conformational heterogeneity of the transporters that sample different sub‐conformations in the inward‐open state without necessarily fully switching to the outward‐open state.^[89]^

Subsequent smFRET studies focused on elucidating the conformational states of MFS transporters during the transport cycle, the timescale of transition between those states, and the influence of substrate and pH.[^89^, ^91^, ^92^, ^163^]

In its apo state, the multidrug efflux protein MdfA from *E. coli* and the multidrug efflux pump LmrP from *Lactococcus lactis* adopt inward‐open and outward‐open states in detergent solution.[^92^, ^105^] The peptide transporter DtpA and the galactoside permease LacY from *E. coli* on the contrary almost exclusively populate inward‐open states under these conditions.[^89^, ^91^] Whereas substrate binding shifted the inward‐open to an outward‐open conformation in LacY,^[91]^ no switch had been observed for DtpA.^[89]^ However, in a lipid environment, DtpA adopted both the outward‐open state and the inward‐open state. Remarkably, the occupation of the different conformations was dependent on the lipid‐type.^[89]^ Interestingly, a “fully‐inward‐open” state was found for DtpA. This state, which shows a wider cytoplasmic opening than expected based on the crystal structure, had not been anticipated by any transport model.^[89]^ Similarly, for LmrP an “extra‐open” state on the periplasmic side and an “inward‐very‐closed” state were observed in detergent.^[105]^ Unfortunately, the role of these additional conformations is currently elusive.

Interestingly, MFS transporters operate at different timescales. In the absence of substrate, MdfA switches conformations with a rate of 0.2 s^−1^ compared to<10 s^−1^ for LmrP.[^92^, ^105^] In both cases, the addition of substrate speeds up the dynamics[^92^, ^105^] but the details had been found to be different. For example, the addition of one substrate but not another, leads to the stabilization of the “extra‐open” state in LmrP.^[105]^ This indicates a transport mechanism that is tailored to the chemical properties of the substrates. Since only one substrate has been tested for LacY^[91]^ and given that DtpA^[89]^ did not show any substrate‐dependent conformational changes, additional studies of MFS transporters are required to confirm this concept.

Most importantly, the studies on DtpA and LmrP revealed that the periplasmic and cytoplasmic side of the transporters are decoupled in detergent,[^89^, ^105^] i. e., the opening of one side of the transporter does not necessarily coincide with closing of the transporters on the other side. In DtpA, this coupling is restored in a lipid environment.^[89]^ Hence, care has to be taken when interpreting results solely based on observations made with detergent solubilized samples.

Finally, it should be mentioned that the conformational state adopted by LmrP and MdfA is influenced by pH. Both proteins preferentially adopt the inward‐open state at low pH[^92^, ^105^] while no such pH‐sensitivity was found for DtpA.^[89]^

In summary, smFRET studies revealed that MFS transporters are highly flexible proteins and our simplistic model of rigid body movements do not fully describe the transport cycle. Moreover, whereas general transport principles are shared among different transporters, dynamics, conformations, and the response to pH changes or substrate additions can differ substantially.

### 3.5 APC superfamily

Besides the MFS superfamily, the amino‐acid‐polyamine‐organocation (APC) superfamily is another large superfamily of secondary active transporters.^[164]^ So far, smFRET experiments were conducted on two subfamilies, the Betaine‐Carnitine‐Choline Transporter (BCCT)^[103]^ and the Neurotransmitter‐Sodium‐Symporters (NSS) family.[^93^, ^94^, ^95^, ^96^, ^110^]

Since the study of BetP (BCCT family) focused mainly on developing smFRET methodology using “caged chromophores”^[103]^ (see Section 2), we will focus on the new findings in the NSS family here.

Secondary active NSS^[165]^ regulate neuron activity by the reuptake of neurotransmitter molecules coupled to physiological sodium gradients, which influence the effect of antidepressants and psychostimulants on the nervous system.^[165]^ To reveal their transport mechanisms, bacterial homologs of NSS were extensively explored, mainly with surface‐immobilized samples using FRET‐coupled TIRF‐microscopy.[^93^, ^94^, ^95^, ^96^, ^110^] These studies provided important details such as a quantitative description of transport cycle steps and substrate‐transporter interactions, which could not be inferred from high‐resolution x‐ray structures or functional studies.^[166]^

Several smFRET studies on LeuT,[^93^, ^94^, ^95^] a single peptide chain transporter with 12 TMs from the thermophilic bacterium *Aquifex aeolicus*, identified conformational variety with opening‐closing dynamics of ∼60 s. The residence times in the outward‐open state were longer in the presence of substrates, thus stabilizing this state and slowing the transport cycle. In addition, Zhao *et al*. unraveled the molecular basis of the specific TM1a helix and its role in the intracellular gating mechanism using various mutations followed by smFRET measurements. Further smFRET studies^[94]^ revealed cooperative allosteric effects between the first binding site of LeuT and the release of the Na^+^ ion in the second binding site of the transporter. The different substrates and ion modulation in solution from Na^+^ to Li^+^ ions, affected the kinetic rates in the transport cycle.

Another smFRET study of LeuT^[95]^ revealed meta‐stable intermediate conformations of the transporter that were hidden in ensemble experiments. These achievements required new photo‐stable dyes that extensively improved the signal‐to‐noise ratio.^[87]^ In addition, the results showed that Na^+^‐binding to the extracellular side of the transporter did not only serve as driving force for transport, but it also provided the required selectivity for a substrate, closed allosterically the intracellular gate, and prevented the premature diffusion of Na^+^ to the intracellular side. Furthermore, the authors showed that open states on both sides of the membrane exist simultaneously, which deviates from the paradigm of the alternating access model.^[69]^

The most recent study of LeuT^[96]^ combines smFRET experiments with MD simulations and imaging techniques. LeVine *et al*. outline the specific amino acids F259 and I359 in the binding pocket and their action as a “volumetric sensor” that dictates different transport rates according to the chemical identity of the substrates (Gly, Ala, Val and Leu). The study revealed that the substrate size affects the rotamer dynamics of a phenylalanine residue (F259) and is associated with the occupancy of the intermediate conformational state (IO2), which in turn controls the intracellular release of Na^+^ that was already proven to be pivotal in the rate determining step.

The amino acid transporter MhsT is an NSS homolog from *Bacillus halodurans* with a LeuT‐like structure^[167]^ and was recently studied with a smFRET substrate sensor.^[110]^ The PBP leucine‐isoleucine‐valine binding protein (LIV‐BP) was used as sensor. LIV‐BP was modified with FRET labels and trapped inside liposomes containing biotinylated MhsT. By insertion of the immobilization tag on either side of the protein, two differently orientated species were engineered. In one sample, the cytoplasmic side of the protein faces the inside of the liposome and in the other, the protein was oriented reversely. A comparison of the smFRET results for the two species showed that MhsT transporters are operating only in the physiological orientation whereas inversely incorporated transporters do not facilitate transport. The first half of the cycle, i. e., substrate binding and substrate release was found to be 0.62±0.08 s^−1^. In the case of the substrate leucine, the second half of the transport cycle, i. e., the return from the apo form to the outward‐open conformation, was found to be the rate‐limiting step with a rate constant of about 1 s^−1^. But in contrast to the current transporter model for NSS transporter dynamics, the second half‐cycle depended on the transported substrate of the first half of the reaction cycle, suggesting the presence of an extracellular allosteric substrate binding site.

Surprisingly, unlike in LeuT, H^+^ ions increased the transport rate in MhsT while Na^+^ lowered it.^[110]^ In conclusion, the influence of different substrates on the transport rate and the dynamics can be diverse within a family. If there is a common pattern within the NSS family, it is still to be discovered.

### 3.6 EAAT family

Similarly to the NSS family, members of the Excitatory‐Amino‐Acid‐Transporters (EAAT) family are also secondary active sodium‐dependent transporters.^[168]^ They are responsible for the uptake of excitatory neurotransmitters from the synaptic cleft and play a key role in the regulation of the nervous system. Understanding their transport mechanisms is therefore key for the development of new psychotherapeutic drugs.^[165]^

The homolog Glt_Ph_ from *Pyrococcus horikoshi* is a suitable model for smFRET experiments because high resolution structures are available for several conformations.[^70^, ^107^, ^169^, ^170^, ^171^, ^172^, ^173^, ^174^, ^175^, ^176^, ^177^, ^178^] These structures revealed a homo‐trimeric assembly and suggest an elevator‐type alternating access mechanism.[^66^, ^69^] Nevertheless, quantitative kinetic data for the transport cycle, its structural basis, the synchronicity between the homo subunit motions around the central trimerization domain, successive substrate uptake, and the influence of a lipid‐mimicking environment remained largely elusive.

Initial smFRET studies^[108]^ aimed at providing information about the transport mechanism and the associated dynamics in presence and absence of substrate of this homo‐trimeric transporter. In these experiments, one subunit carried a biotin moiety whereas the other two subunits were labeled with a FRET dye pair. TIRF imaging showed that the trimerization domain was devoid of significant dynamics. However, the individual monomers remained mobile in the trimer and the complex preferentially adopted an outward‐open state. In agreement with the proposed elevator‐like mechanism, also the inward‐open conformer and an intermediate were sampled. Point mutations based on the known crystal structure caused dramatic changes in these populations and supported the proposed mechanism. Interestingly, substrate addition not only shifted the occupation of different conformers but it also decelerated the interconversion between these states. Importantly the single‐molecule trajectories revealed that the switching between conformations might be rather fast or slow for prolonged periods. Periods of fast and slow switching but also non‐dynamic periods were found in the apo state. Upon substrate addition, mostly the dynamic periods prevailed. Further smFRET studies with surface‐immobilized liposomes^[107]^ in combination with x‐ray crystallography suggested a mechanism based on presence and absence of locked configurations, which is responsible for the rapid and slow dynamic periods. The authors compared wild type transporter with its “humanized form” variant R276S/M395R (H_276_,_395_‐Glt_Ph_). Ensemble experiments with this variant but also with the human transporter showed faster dynamics compared to the wild type protein.^[179]^ However, the smFRET study revealed that the quiescent periods, which are the hallmark of wild type Glt_Ph_, were absent in the H_276_,_395_−Glt_Ph_ variant. In addition, smFRET histograms showed that the locked configurations, which are responsible for the quiescent periods, were disrupted in H_276_,_395_−Glt_Ph_. The inward‐open states of the variant showed an increased donor‐acceptor distance compared to wild type, suggesting an enlargement of the binding pocket in the inward‐open state, which was confirmed using x‐ray crystallography and molecular dynamics simulations.

Another smFRET investigation^[114]^ of Glt_Ph_ studied the synchronicity between the movements of the homo subunits of the transporter in a vesicle environment. Erkens *et al*. explored the dynamics that create the symmetric outward‐facing state^[169]^ and the asymmetric state in which one of the subunits is in the inward‐open position^[70]^ whereas the others are in outward‐open states. The triply labeled species, ‘two‐donor‐one‐acceptor’ and ‘one‐donor‐two‐acceptor’, demonstrated a complex trajectory, revealing that the subunits of a single trimer might independently populate different conformations.^[114]^ An analytical model assuming unsynchronized conformational transitions of individual subunits reproduced the shape of the dwell‐time distributions. The most recent smFRET study^[106]^ on Glt_Ph_ monitored aspartate‐uptake in liposomes using an L‐aspartate‐binding FRET sensor. Surprisingly, uptake rates for single molecules were found to be preserved over multiple uptake cycles, thus exhibiting “molecular memory”, i. e., transporters with slow rates and transporters with fast rates preserve their speed over successive uptake cycles.^[106]^ This phenomena can be observed only by single molecule techniques.^[180]^ Further studies are necessary to elucidate the molecular basis for this phenomenon.

### 3.7 SMR family

Another family of secondary active transporters studied by smFRET is the small multidrug resistance (SMR) family. Members of this family are found in prokaryotes where they utilize an electrochemical proton gradient to expel toxic substances such as quaternary ammonium compounds from the cell.[^182^, ^183^] Transporters belonging to the SMR family are typically multimers. A single subunit is 100–140 amino acids in length and contains four TMs (Figure 4).^[183]^ A particularly well‐characterized member of this family is the multidrug efflux protein EmrE from *E. coli*. This transporter functions as an antiporter that removes drug molecules from the cell via a coupling to the import of protons.^[184]^ It was already established that the functional unit is a dimer but the orientation of the dimer in the membrane is still under debate.[^185^, ^186^, ^187^, ^188^, ^189^, ^190^] Using smFRET, NMR spectroscopy, and crosslinking, Morrison *et al*. were able to show that EmrE is organized as an antiparallel dimer in which both subunits transport the substrate at the same time but in opposite directions.^[181]^ Interestingly, smFRET experiments do not show fluctuations between two states although NMR experiments established that the transporter switches between different conformations.^[181]^ This agrees with the model of an antiparallel dimer in which both monomers change conformation simultaneously such that the distance between the FRET labels remains unchanged.

### 3.8 CLC family

The chloride‐carrier‐channel (CLC) family consists of ion channels and transporters. SmFRET studies of a CLC transporter (CLC‐ec1 from *E. coli* ^[191]^) became available just recently.^[104]^ CLC transporters are secondary active anion/proton exchangers in the majority of intracellular membranes where they regulate ion concentrations. They are functional dimers where each unit takes up Cl^−^ ions and simultaneously exports H^+^ to the environment^[192]^ in a stoichiometric 2 : 1 ratio.[^191^, ^193^] It was postulated that the individual subunits work independently of each other^[194]^ and smFRET was used to test this hypothesis. To ensure the observation of one subunit at a time, the two subunits were expressed separately, one carrying a twin‐strep tag for surface‐immobilization and the other subunit carrying a FRET dye pair. In addition, the subunit that carried the surface‐immobilization tag was locked in an inactive conformation (QQQ‐variant). Transporters with one inactive subunit and one FRET labeled subunit were immobilized while dimers with two FRET labeled subunits did not bind.^[104]^ By using an inactive variant that mimicked the protonated monomer (QQQ‐variant) known to adopt the outward‐open state,^[115]^ transitions from the outward‐open to occluded state of a transporter unit could be observed. Remarkably, spatial changes associated with this transition were only in the range of 1–2.5 Å and were independent of whether the second monomer was active or inactive. This result indeed confirmed the lack of cooperativity between the subunits.^[104]^

### 3.9 SWEET family

Unlike the previously described families of primary and secondary active transporters, members of the Sugar‐Will‐Eventually‐be‐Exported‐Transporters (SWEET) family do not require an additional energy source for transport. SWEETs facilitate the transport of mono‐ and disaccharides along the concentration gradient.[^195^, ^196^] These transporters are proposed to function according to the alternating access rocker‐switch mechanism,[^66^, ^197^] but at the same time, the substrate selectivity, recognition, and the cooperative function of oligomers is poorly understood.

A recent x‐ray study combined with smFRET measurements and functional characterizations of AtSWEET13 from *A. thaliana* provided new insights.^[78]^ The x‐ray structure of the inward‐open transporter identified substrate binding residues and explained why sucrose is bound tighter than glucose, a finding that was further supported by smFRET.^[78]^ The smFRET measurements further outlined the relative positions of the different helices in the apo and substrate‐bound states, thus proposing a gating mechanism for the protein monomers. While the x‐ray structure for AtSWEET13 showed a monomeric assembly in the crystal lattice, smFRET data and other biophysical methods supported dimer formation. Based on further smFRET experiments, a cooperative “revolving door” model, was proposed. According to this model the protein acts as a dimer in which a conformational transition of the substrate‐carrying protomer is coupled to the substrate‐free opposite transition in the other monomer.^[78]^

## 4 Concluding Remarks

With the recent advances of high‐resolution techniques,[^198^, ^199^] the number of membrane transporter structures is steadily increasing. Yet, structures only provide partial information about the mechanism and dynamics of transport. Moreover, it is challenging and sometimes impossible to estimate the conformational variety of these biomolecules based on static x‐ray or cryo‐EM structures alone.

SmFRET has been shown to complement such structural data with information on alternative conformers and their associated timescales of interconversion. This is a key advantage, not only compared to x‐ray crystallography or cryo‐EM, but also to other ensemble biophysical measurements.

To understand transport mechanisms on a molecular basis, data interpretation of smFRET experiments can highly benefit from available high‐resolution structures, combined with MD simulations, mass spectrometry, SAXS and NMR studies. The power of combining smFRET studies with NMR spectroscopy has already been demonstrated on model systems such as EmrE and members of the glutamate transporter family.[^174^, ^181^, ^200^] The techniques are highly complementary and we expect that combining the residue‐level information provided by solution and solid‐state NMR with the ability of smFRET to resolve conformational and dynamic heterogeneity in molecule ensembles will be key in future attempts to understand the mechanisms of membrane transporters. In addition, since smFRET became a broadly accessible method over the past two decades, we envision that it will also guide future approaches for obtaining high‐resolution structures of distinct conformational states as it can serve as a rapid screening technique to identify suitable conditions (pH, substrate, buffer, mutations etc.) where the desired conformational state is the predominant population.

In this mini‐review, we discussed recent smFRET results on membrane transporters and highlighted their contributions to advance the mechanistic understanding of this protein class. Technical developments in the field of smFRET spectroscopy, including available commercial setups and the fact that multiple protocols and tools for labeling proteins with fluorescent dyes are available, suggest that this method will evolve to a standard approach for investigating membrane proteins in the future. We expect that it will be possible to record full transport cycles of diverse transporters at a single‐molecule level and to deduce their kinetic characteristics. Such setups not only open the door to understand the impact of different lipids on transport rates and conformational dynamics but they will also be essential in elucidating the function of transporter binding proteins, which have recently been discovered.[^201^, ^202^]

## Conflict of interest

The authors declare no conflict of interest.

## Biographical Information

*Kim Bartels studied chemistry and biochemistry at the Ludwig Maximilian University Munich (Germany). During her studies she worked on the structure determination of bacterial transmembrane proteins under the supervision of Dr. K. Beis (Imperial College London). After receiving her MSc degree in biochemistry, she joined the group of Christian Löw at EMBL Hamburg as a PhD student in 2016. Here, she was using single molecule FRET to study the structural dynamics of proton‐dependent oligopeptide transporters*.

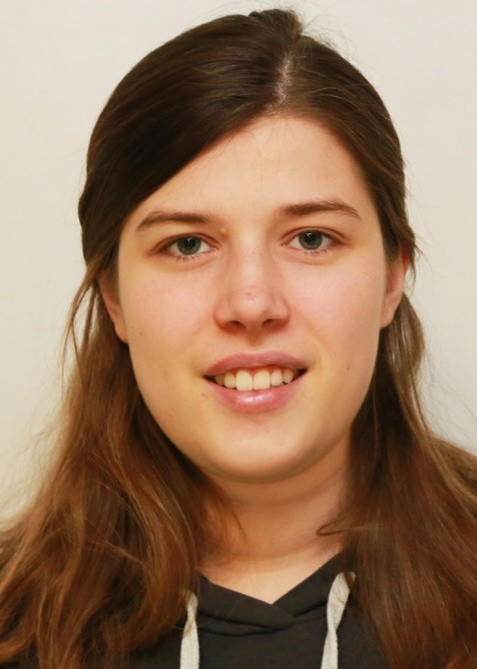

## Biographical Information

*Tanya (Tatyana) Lasitza Male studied B.Sc. in Chemistry in Tel‐Aviv University (Israel). Afterwards she continued to M.Sc. in the Department of Physical Chemistry (Tel‐Aviv University). Her M.Sc project was guided by Dr. Eli Stern and Prof. Dan Huppert She is currently studying for her Ph.D. in the Department of Chemical and Structural Biology of the Weizmann Institute of Science (Israel), under the supervision of Dr. Hagen Hofmann. She is specializing in single‐molecule fluorescence spectroscopy and dynamics of membrane proteins*.

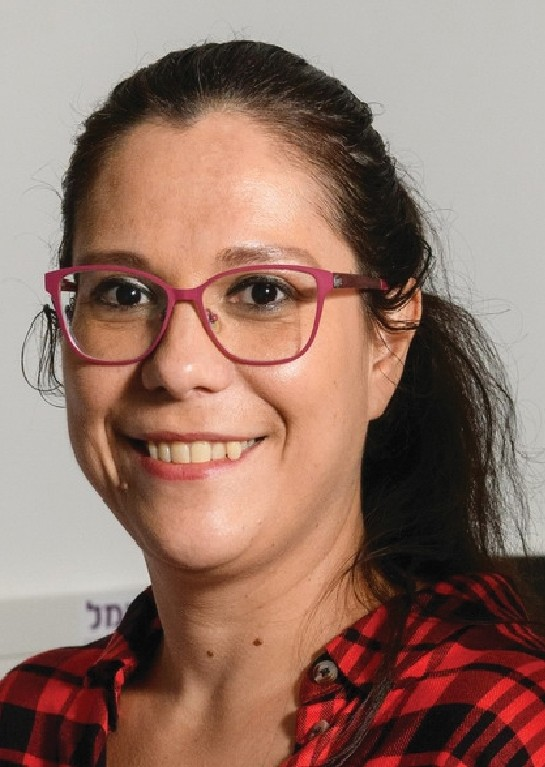

## Biographical Information

*Hagen Hofmann (born 1978) studied biochemistry and received his PhD from the Martin‐Luther University Halle‐Wittenberg (Germany). He did his post‐doctoral research in single‐molecule biophysics at the University of Zurich (Switzerland). He then joined the Department of Chemical and Structural Biology of the Weizmann Institute as group leader, where he is working on the dynamics of intrinsically disordered proteins and protein complexes, especially using single‐molecule fluorescence spectroscopy*.

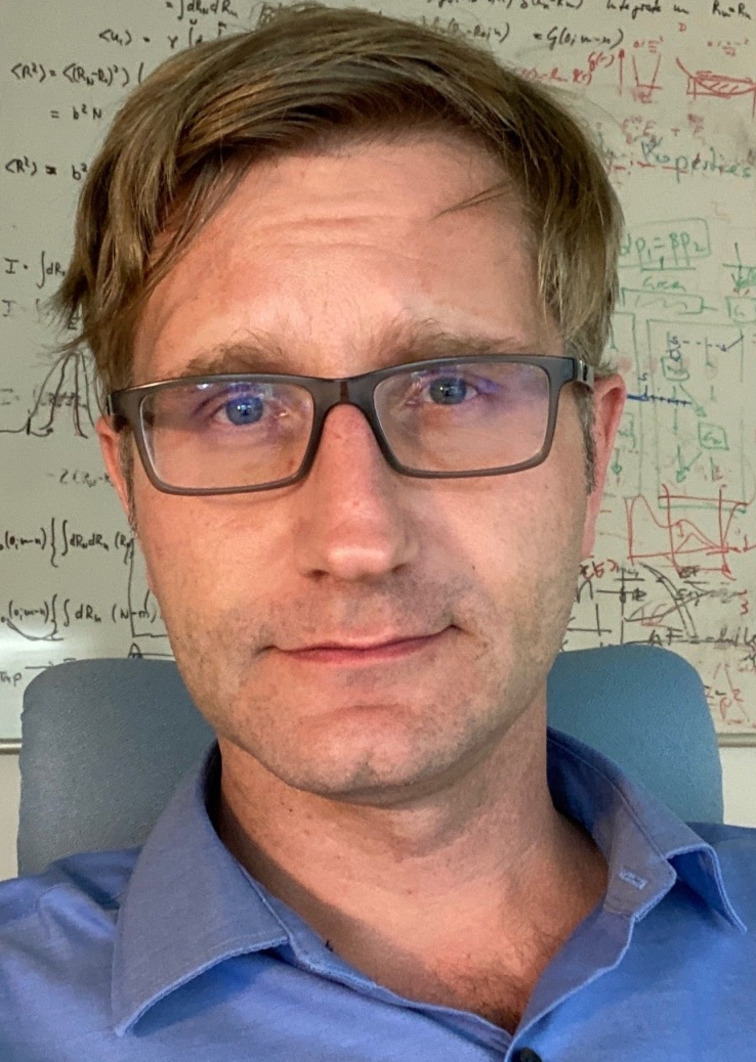

## Biographical Information

*Christian Löw (born 1979) studied biochemistry at the University of Bayreuth (German) and received his PhD from the Martin‐Luther University Halle‐Wittenberg (Germany). He did his post‐doctoral research in membrane protein structural biology at the Karolinska Institute in Stockholm (Sweden). He then moved as group leader to the European Molecular Biology Laboratory (EMBL) in Hamburg and joined the Centre for Structural Systems Biology (CSSB), where he is studying integral membrane nutrient transporters, mainly using structural and biochemical approaches*.

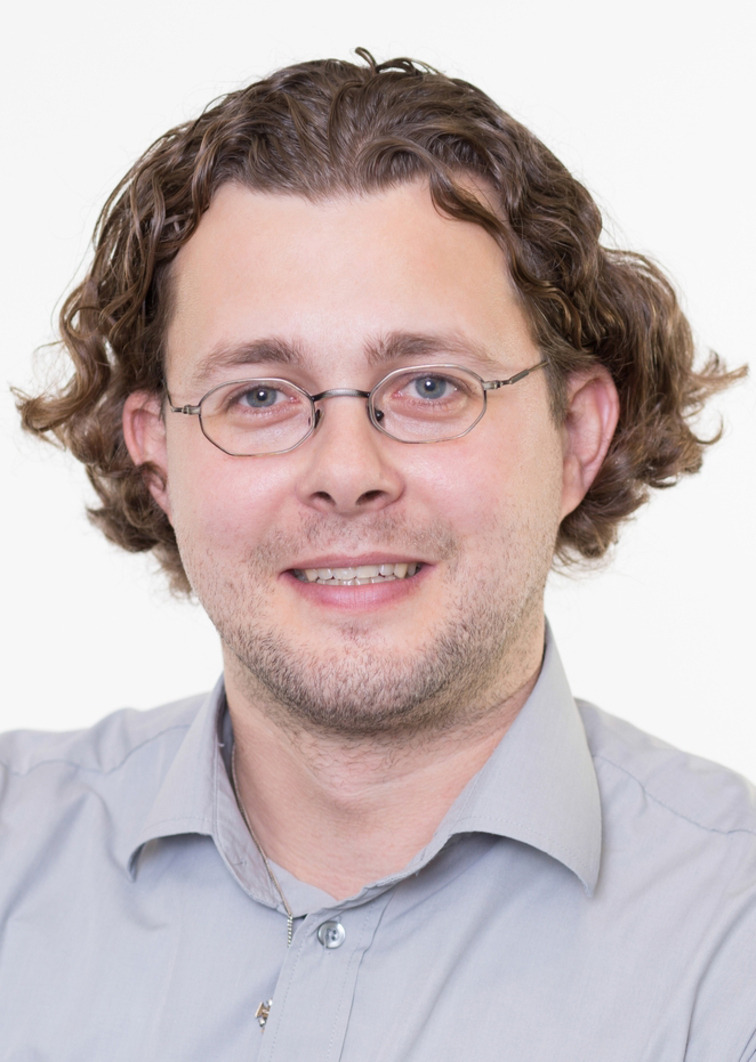
